# Transcutaneous carbon dioxide suppresses skeletal muscle atrophy in a mouse model of oral squamous cell carcinoma

**DOI:** 10.1371/journal.pone.0302194

**Published:** 2024-04-17

**Authors:** Aki Sasaki, Daisuke Takeda, Hotaka Kawai, Yoshiaki Tadokoro, Aki Murakami, Nanae Yatagai, Satomi Arimoto, Hitoshi Nagatsuka, Masaya Akashi, Takumi Hasegawa

**Affiliations:** 1 Department of Oral Maxillofacial Surgery, Kobe University Graduate School of Medicine, Japan; 2 Department of Oral Pathology and Medicine, Graduate School of Medicine, Dentistry and Pharmaceutical Sciences, Okayama University, Okayama, Japan; Cinvestav-IPN, MEXICO

## Abstract

Cancer cachexia causes skeletal muscle atrophy, impacting the treatment and prognosis of patients with advanced cancer, but no treatment has yet been established to control cancer cachexia. We demonstrated that transcutaneous application of carbon dioxide (CO_2_) could improve local blood flow and reduce skeletal muscle atrophy in a fracture model. However, the effects of transcutaneous application of CO_2_ in cancer-bearing conditions are not yet known. In this study, we calculated fat-free body mass (FFM), defined as the skeletal muscle mass, and evaluated the expression of muscle atrophy markers and uncoupling protein markers as well as the cross-sectional area (CSA) to investigate whether transcutaneous application of CO_2_ to skeletal muscle could suppress skeletal muscle atrophy in cancer-bearing mice. Human oral squamous cell carcinoma was transplanted subcutaneously into the upper dorsal region of nude mice, and 1 week later, CO_2_ gas was applied to the legs twice a week for 4 weeks and FFM was calculated by bioimpedance spectroscopy. After the experiment concluded, the quadriceps were extracted, and muscle atrophy markers (muscle atrophy F-box protein (MAFbx), muscle RING-finger protein 1 (MuRF-1)) and uncoupling protein markers (uncoupling protein 2 (UCP2) and uncoupling protein 3 (UCP3)) were evaluated by real-time polymerase chain reaction and immunohistochemical staining, and CSA by hematoxylin and eosin staining. The CO_2_-treated group exhibited significant mRNA and protein expression inhibition of the four markers. Furthermore, immunohistochemical staining showed decreased MAFbx, MuRF-1, UCP2, and UCP3 in the CO_2_-treated group. In fact, the CSA in hematoxylin and eosin staining and the FFM revealed significant suppression of skeletal muscle atrophy in the CO_2_-treated group. We suggest that transcutaneous application of CO_2_ to skeletal muscle suppresses skeletal muscle atrophy in a mouse model of oral squamous cell carcinoma.

## Introduction

Cancer cachexia is a multifactorial syndrome defined by an ongoing loss of skeletal muscle mass (with or without loss of fat mass) that cannot be fully reversed by conventional nutritional support and leads to progressive functional impairment. Its pathophysiology is characterized by a negative protein and energy balance driven by a variable combination of reduced food intake and abnormal metabolism [1, 2]. Cancer cachexia has been defined in various ways, but the most prominent characteristic is skeletal muscle atrophy. Cancer cachexia is quite common in patients with advanced cancer [3, 4]. Furthermore, it is a cause of resistance to chemotherapy and radiotherapy and is attributed to at least 20% of deaths in cancer patients [5, 6]. However, there is currently no established treatment for cancer cachexia, and skeletal muscle atrophy is the most significant clinical symptom [3, 5].

Skeletal muscle mass is maintained by a balance between protein synthesis and proteolysis, and if this balance shifts to a catabolic state, it leads to skeletal muscle atrophy [6]. Skeletal muscle atrophy is regulated by the ubiquitin–proteasome pathway, specifically the rate-limiting enzymes of MAFbx (muscle atrophy F-box protein) and MuRF-1 (muscle RING-finger protein 1). Furthermore, several in vivo studies suggested that these factors are increased due to skeletal muscle atrophy [7–11].

Skeletal muscle atrophy is also related to a decrease in ATP synthesis. This is thought to be regulated by uncoupling proteins (UCPs), which are recognized as transporters of proton ions between the mitochondrial inner membrane and the mitochondrial matrix. There are five known isoforms of UCP (UCP1 to UCP5). UCP2 is present in all tissues, including skeletal muscle, while UCP3 is expressed in skeletal and cardiac muscle [12]. Several in vivo studies suggested that UCP2 and UCP3 are related to skeletal muscle atrophy [12–15].

Carbon dioxide (CO_2_) therapy is widely known as an effective treatment to improve blood flow and has been indicated for various diseases such as heart disease and skin conditions [16]. We demonstrated that transcutaneous application of CO_2_ could improve local blood flow and promote the suppression of muscle atrophy and contraction, as well as the recovery of muscle damage [17–23]. However, while we have demonstrated the effect of transcutaneous application of CO_2_ in suppressing tumor growth [24], we have not demonstrated the effect of cancer cachexia. Therefore, we hypothesized that transcutaneous CO_2_ application suppresses skeletal muscle atrophy in oral cancer-bearing mice. In this study, we investigated the relationship of skeletal muscle mass and the expression of MAFbx, MuRF-1, UCP2, and UCP3 in the CO_2_-treated group.

## Materials and methods

### Cell culture

In this study, we obtained an oral cancer cell line, HSC-3, from the Health Science Research Resources Bank (Osaka, Japan). This cell line originates from a metastatic deposit of a poorly differentiated squamous cell carcinoma (SCC) of the tongue, specifically in a mid-internal jugular lymph node, extracted from a 64-year-old man. The cultivation of HSC-3 cells was performed using Eagle’s minimum essential medium (Sigma–Aldrich, St. Louis, MO, USA), which was supplemented with fetal bovine serum (10%; Thermo Fisher Scientific, Waltham, MA, USA), as well as a solution of penicillin and streptomycin (1000 units/mL; Sigma–Aldrich). Trypsin (0.25%; FUJIFILM Wako Pure Chemical Corporation, Osaka, Japan) and ethylenediaminetetraacetic acid (0.02%; DOJINDO LABORATORIES, Kumamoto, Japan) solutions were used to isolate cells for subculture, as previously described [24].

### Animal models

We obtained 7-week-old male athymic BALB/cAJcl–nu/nu nude mice from CLEA Japan (Tokyo, Japan). These mice were maintained in a pathogen-free environment by trained staff, strictly adhering to institutional protocols. The animal experiments were approved by the Institutional Animal Care and Use Committee (permission number: P-210105) and were performed in accordance with the Guidelines for Animal Experimentation at Kobe University Animal Experimentation Regulations. We injected HSC-3 cells (2.0×10^6^ cells in 500 μL Eagle’s minimum essential medium per mouse) subcutaneously into the upper dorsal region of the mouse under anesthesia using isoflurane, in accordance with previous studies of animal models of cancer cachexia [25–28].

### Measurement of fat-free body mass by bioimpedance spectroscopy

In vivo body composition was measured by bioimpedance spectroscopy (ImpediVet; ImpediMed, Carlsbad, CA, USA) once a week for 4 weeks. The mice underwent weighing, followed by anesthesia induction using a mixed anesthetic solution comprising medetomidine, midazolam, and butorphanol. The anesthesia was administered through intraperitoneal injection, after which the mice were positioned in a prone stance on a table. Four 25G needles, functioning as electrodes, were positioned subcutaneously along the midline of the back in accordance with the instructions provided by the manufacturer. The total body water was quantified and utilizing the differing water composition of adipose and lean tissues, an approximation of the overall fat mass and fat-free body mass (FFM) was calculated. The skeletal muscle mass was defined as the FFM. FFM (kg) was calculated every week starting from the tumor transplantation day until the end of the experiment. During each measurement, we calculated the percentage of FFM relative to the individual’s body weight and tracked its changes over time, using the day of tumor transplantation as the reference point.

### Transcutaneous CO_2_ treatment

**Fig 1:**
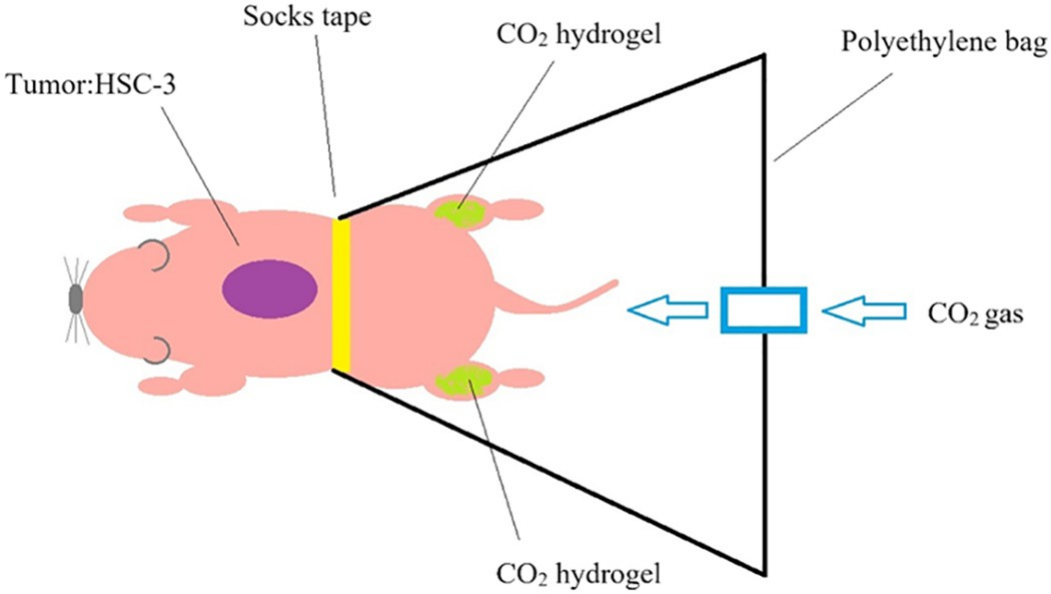
Transcutaneous CO_2_ treatment.

The skin surface of the legs, excluding the tumor implant site, was covered with a CO_2_ absorption-enhancing hydrogel (CO_2_ hydrogel) obtained from CO_2_ Tech (Kobe, Japan). This area was sealed with a polyethylene bag to retain the gas inside (Fig 1). Then 100% CO_2_ gas was pumped into the bag and transcutaneous CO_2_ treatment was applied for 20 min. Upon completion of the CO_2_ treatment duration, the CO_2_ hydrogel was gently removed from the treated area. Control animals were treated similarly, with room air replacing the CO_2_ [18, 23, 24].

The skin surface of the legs, excluding the tumor implant site, was covered with a CO_2_ absorption-enhancing hydrogel (CO_2_ hydrogel). 100% CO_2_ gas was pumped into the bag and transcutaneous CO_2_ treatment was applied for 20 min.

### In vivo

Fourteen mice were randomly assigned to two groups: a CO_2_-treated group (n = 7) and a control group (n = 7). The treatment was initiated at 7 days following the implantation of HSC-3 cells and was administered twice a week for 4 weeks. Referring to our previous studies, the endpoint was set at 4 weeks after implantation [24]. Tumor volume was calculated as previously described according to the formula V = π/6×a^2^×b, where a and b represent the shorter and longer diameters of the tumor, respectively [24].

### Surgical procedure

The treatment protocol spanned a duration of 4 weeks from its initiation, and upon its completion, 24 hours after the final treatment session, the mice were humanely sacrificed under anesthesia. No animals died before euthanization. Mice were adequately anesthetized by inhalation with isoflurane and compassionately killed by decapitation to remove blood. Subsequently, both the tumor and bilateral quadriceps muscles were extracted, and we harvested the bilateral quadriceps muscles from mice. One was used for the RNA extraction, and the other was used for hematoxylin and eosin (H&E) and immunohistochemical (IHC) staining.

### Quantitative real-time polymerase chain reaction

To analyze the mRNA expression levels of *MAFbx*, *MuRF-1*, *UCP2*, and *UCP3* in the quadriceps muscles of both the control and CO_2_-treated groups, quantitative real-time PCR was employed. The treated quadriceps muscles for quantitative real-time PCR were processed for total RNA extraction according to the manufacturer’s guidelines using a RNeasy Mini Kit (Qiagen, Valencia, CA, USA), which involves selective binding to a silica gel-based membrane [24]. cDNA was synthesized from a total of 300 ng RNA using the High-Capacity cDNA Transcription kit from Applied Biosystems (Foster City, CA, USA). The quantification of mRNA transcripts was executed using the Applied Biosystems StepOne Real-Time PCR System (Applied Biosystems). Real-Time PCR reactions (20 μL) contained 0.5 μM forward primer, 0.5 μM reverse primer, and 1 μL cDNA template from the RT reaction, and 10 μL (2×) Power SYBR green master mix (Applied Biosystems). Reaction conditions were 95°C for 10 min, followed by 40 cycles of 95°C for 15 sec and 60°C for 1 min. The level of each target gene was normalized to *ACTB* levels and expressed relative to the levels of the control group (^ΔΔ^CT method; Applied Biosystems) [19, 24]. The primer sequences purchased from Thermo Fisher Scientific are described in Table 1 [10].

**Table 1:** Specific primer sequences for real‐time polymerase chain reaction analysis.

Gene name	Primer sequence (5’-3’)	
ACTB	Fw: CTG GCT CCT AGC ACC ATG AA	Rv: CTG CTT GCT GAT CCA CAT CT
MAFbx	Fw: TTA TGC ACG CTG GTC CAG A	Rv: TGT AAG CAC ACA GGC AGG TC
MuRF-1	Fw: GGT GCC TAC TTG CTC CTT GT	Rv: TCA CCT GG TGG CTG TTT TC
UCP2	Fw: TGT AAG CAC ACA GGC AGG TC	Rv: CAT GGT CAG GGC ACA GTG GC
UCP3	Fw: GTG ACC TAT GAC ATC ATC AAG GA	Rv: GCT CCA AAG GCA GAG ACA AAG

### Hematoxylin and eosin (H&E) staining and analysis of the cross-sectional area of muscle

The quadriceps muscles were treated for H&E staining without detaching from the femur, to use the femur the referencing the gross position, by fixation in 4% paraformaldehyde and embedding in paraffin wax. We generated a 3-μm-thick section of the quadriceps muscle with the midpoint of the femur using a microtome and stained with H&E. The cross-sectional area of the muscle was compared between groups. Section images were captured randomly using a BZ-X700 microscope at ×400 magnification (KEYENCE, Osaka, Japan) and the cross-sectional area was measured using a BZ-X700 Analyzer (KEYENCE) [18].

### Immunohistochemical staining (IHC) and quantification of protein expression

IHC was performed using the antibodies outlined in Table 2. The sections were subjected to deparaffinization using a series of xylene treatments lasting 15 min each, followed by rehydration through graded ethanol solutions. Endogenous peroxidase activity was blocked by incubating the sections in a solution of 0.3% H_2_O_2_ in methanol for a duration of 30 min. Subsequent to antigen retrieval, the sections were subjected to treatment with 10% normal serum for 15 min, followed by incubation with primary antibodies overnight at a temperature of 4°C. The signals were amplified using the avidin–biotin complex method (Vector Lab, Burlingame, CA, USA). Color development was achieved using DAB (Nakalai Tesque, Kyoto, Japan), and the sections were counterstained with Mayer’s hematoxylin. The staining outcomes were visualized using an all-in-one fluorescence microscope (BZ-X700; KEYENCE) [19]. Three locations were arbitrarily selected from one sample at ×400 magnification and the immunohistochemical staining ratio of muscle fibers that were judged DAB positive was quantified. The same work was performed for all samples and the average value was calculated for each sample.

**Table 2:** Primary antibodies used in IHC.

Primary antibody	Immunized animal	Antigen retrieval	Dilution	Supplier
MAFbx	Mouse	microwave heating in Dako Target Retrieval Solution, pH9(×10) at 100°C for three and a half minutes	1:1200	ProteinTech Group, Chicago, IL
MuRF-1	Rabbit	microwave heating in Dako Target Retrieval Solution, pH9(×10) at 100°C for three and a half minutes	1:500	ProteinTech Group, Chicago, IL
UCP2	Rabbit	microwave heating in Dako Target Retrieval Solution, pH9(×10) at 100°C for three and a half minutes	1:400	Bioss Antibodies, Boston, MA, United States
UCP3	Rabbit	microwave heating in Dako Target Retrieval Solution, pH9(×10) at 100°C for three and a half minutes	1:200	Novus Biologicals LLC, Centennial, Colorado, United States

### Statistical analysis

Data are presented as the average ±standard deviation. EXCEL Toukei Ver. 7.0 for Windows (ESUMI Co., Ltd., Tokyo, Japan) was used for the statistical analyses performed on the data for two groups by the Mann–Whitney U test. The level of statistical significance was set at P<0.05.

## Results

### 1. FFM (defined as the skeletal muscle mass)

**Fig 2:**
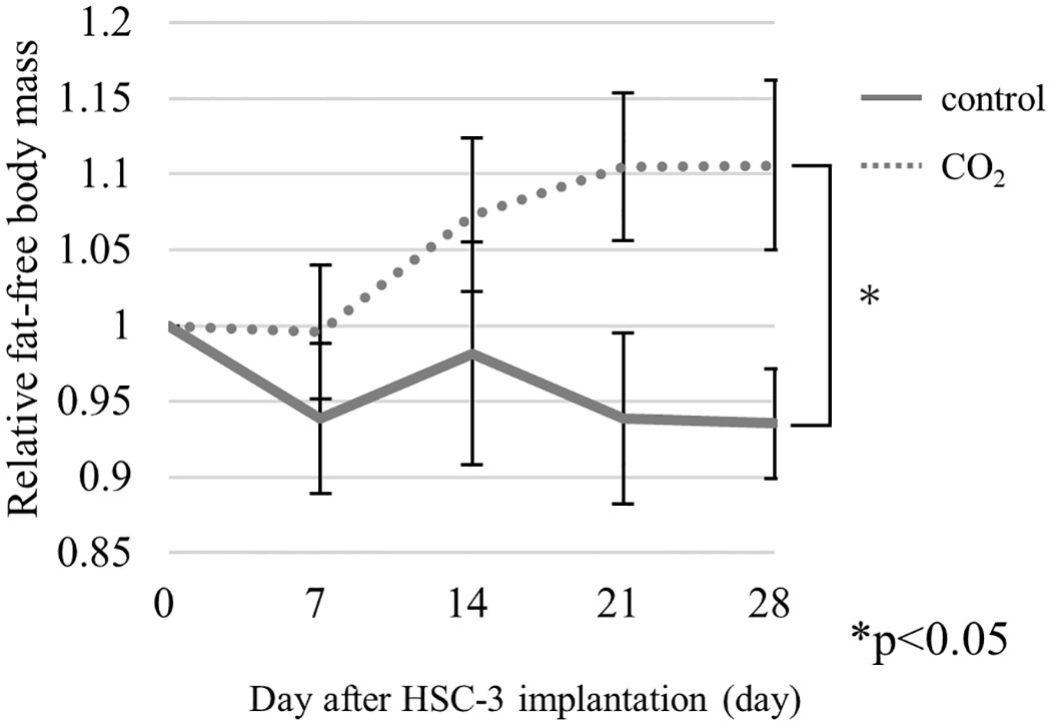
The relative FFM, defined as skeletal muscle mass in the control group (n = 7) and CO_2_-treated group (n = 7) (*P<0.05). At the end of the experiment, FFM (average ± standard deviation) of the control group was 0.94 ± 0.10, and that of the CO_2_-treated group was 1.11± 0.15.

At 7 days after HSC-3 implantation, CO_2_ gas was applied to the legs twice a week for 4 weeks. We investigated the changes in FFM over time relative to the baseline at the beginning of the measurement. At the end of the experiment, FFM (average ± standard deviation) of the CO_2_-treated group was 1.11± 0.15, and that of the control group was 0.94 ± 0.10. Furthermore, FFM had decreased by significantly more in the control group than in the CO_2_-treated group (*P<0.05) (Fig 2).

### 2. Tumor volume

**Fig 3:**
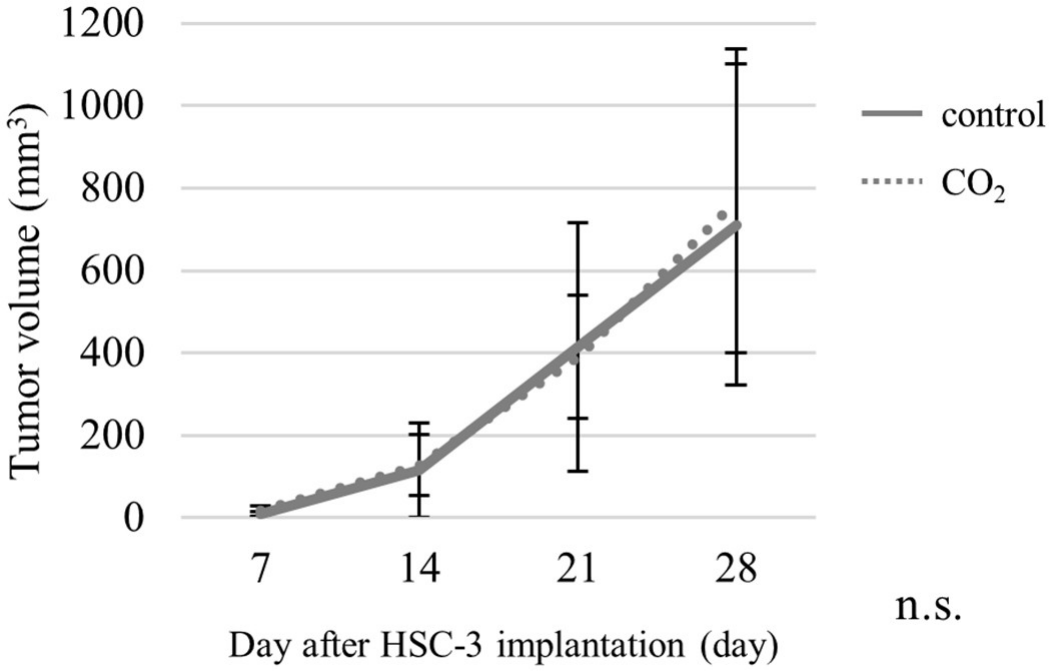
Tumor volumes in the control group (n = 7) and CO_2_-treated group (n = 7).

At any point during the measurement of tumor volume, the differences in the tumor volume were not statistically significant in the control and CO_2_-treated groups (Fig 3).

### 3. Gene expression

**Fig 4:**
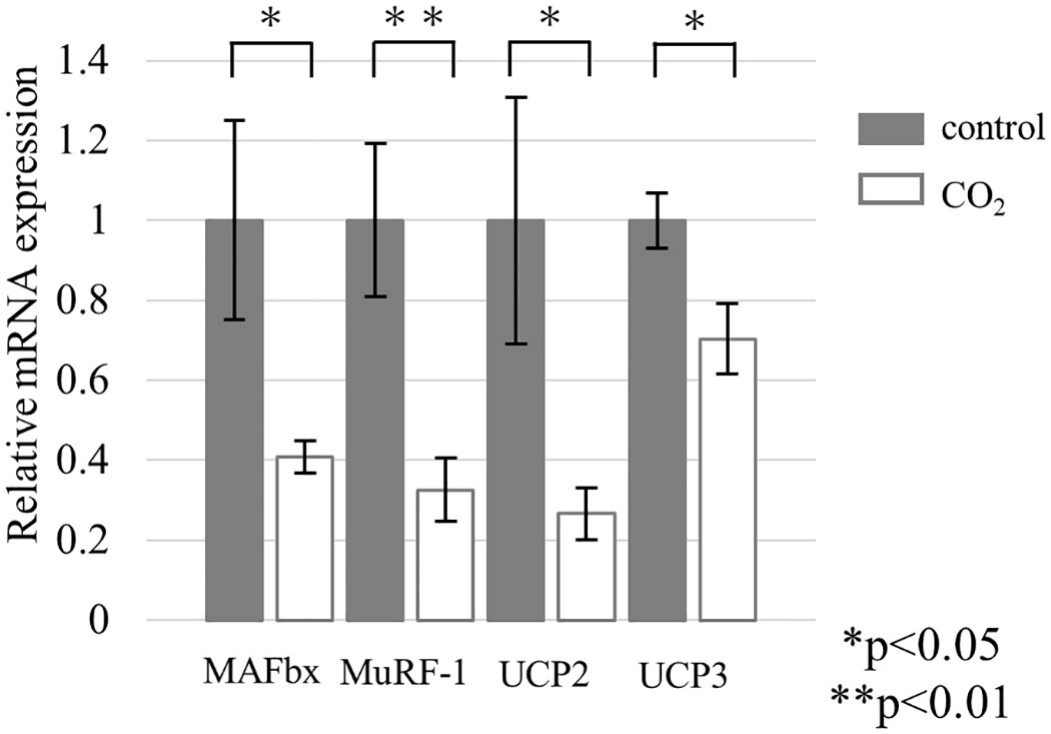
Relative mRNA expression in the control group (n = 7) and CO_2_-treated group (n = 7) (*P<0.05, **P<0.01).

At the end of the experiment, quantitative real-time PCR demonstrated that the mRNA expression levels of *MAFbx*, *MuRF-1*, *UCP2*, and *UCP3* were significantly suppressed in the CO_2_-treated group compared with the control group (*P<0.05, **P<0.01) (Fig 4). At the end of the experiment, average *MAFbx* expression levels (± standard deviation) in the control group were 1.00±0.66, and those in the CO_2_-treated group were 0.41±0.11. *MuRF-1* expression levels in the control group were 1.00±0.51, and those in the CO_2_-treated group were 0.32±0.21. *UCP2* expression levels in the control group were 1.00±0.82, and those in the CO_2_-treated group were 0.27±0.17. *UCP3* expression levels in the control group were 1.00±0.18, and those in the CO_2_-treated group were 0.70±0.23.

At the end of the experiment, average *MAFbx* expression levels (± standard deviation) in the control group were 1.00±0.66, and those in the CO_2_-treated group were 0.41±0.11. *MuRF-1* expression levels in the control group were 1.00±0.51, and those in the CO_2_-treated group were 0.32±0.21. *UCP2* expression levels in the control group were 1.00±0.82, and those in the CO_2_-treated group were 0.27±0.17. *UCP3* expression levels in the control group were 1.00±0.18, and those in the CO_2_-treated group were 0.70±0.23.

### 4. Histological analysis

**Fig 5:**
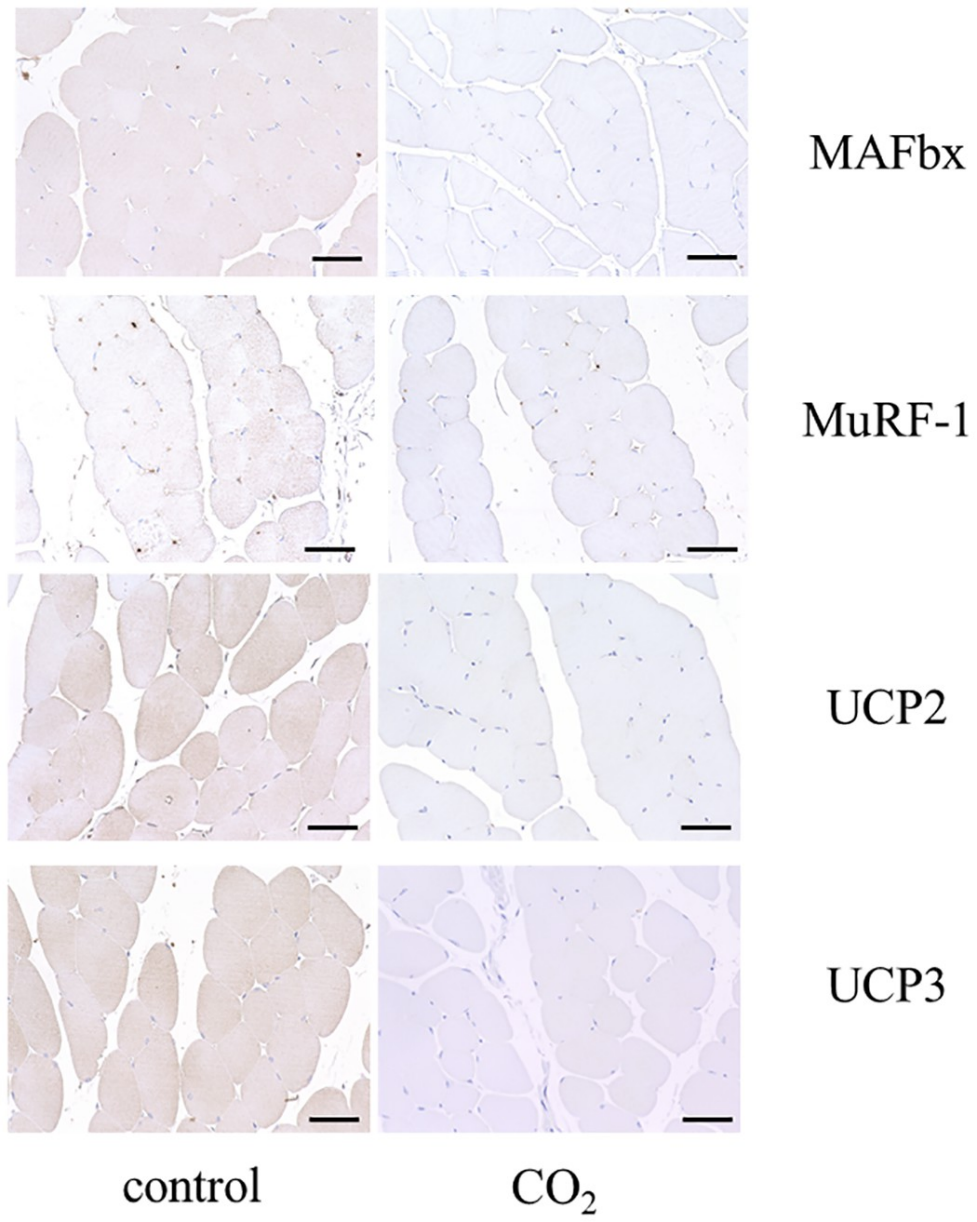
Immunohistochemical staining for MAFbx, MuRF-1, UCP2, and UCP3 in the control group and CO_2_-treated group. The representative images are shown at ×400 magnification. Scale bar = 50 μm.

**Fig 6:**
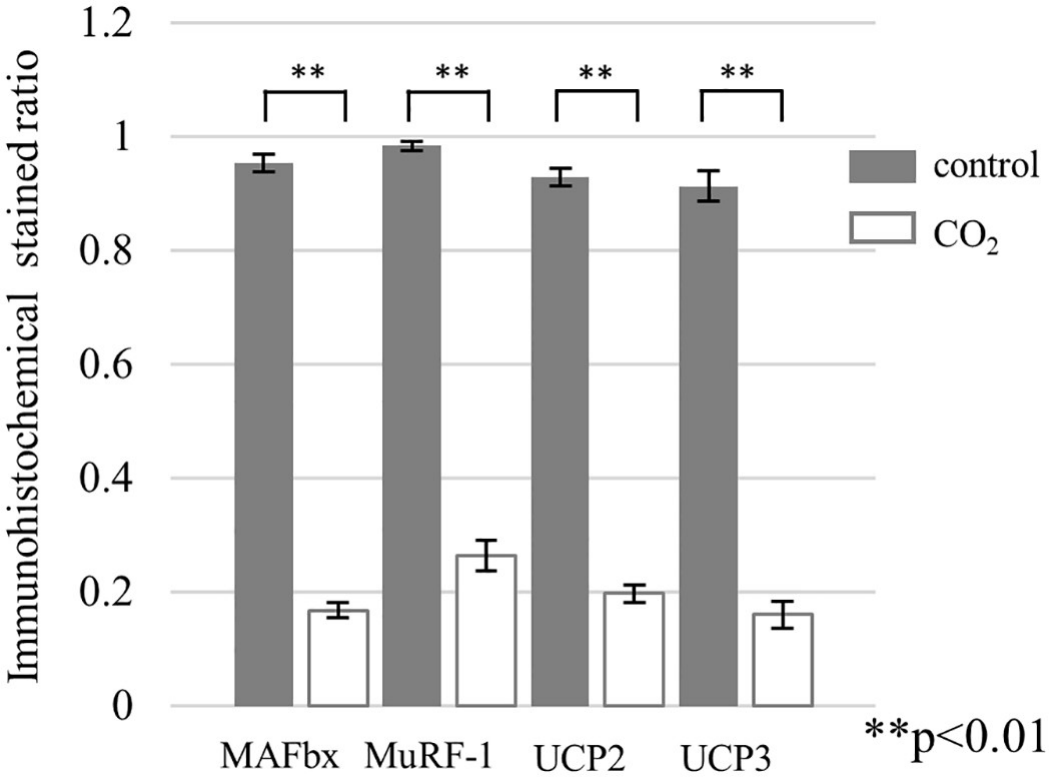
Immunohistochemical staining ratio for MAFbx, MuRF-1, UCP2, and UCP3 in the control group (n = 7) and CO_2_-treated group (n = 7) (**P<0.01). MAFbx expression (± standard deviation) in the control group was 0.95±0.04, and that in the CO2-treated group was 0.17±0.04. MuRF-1 expression in the control group was 0.98±0.02, and that in the CO2-treated group was 0.26±0.07. UCP2 expression in the control group was 0.93±0.04 and that in the CO2-treated group was 0.20±0.11. UCP3 expression in the control group was 0.91±0.07, and that in the CO2-treated group was 0.16±0.06.

At the end of the experiment, immunohistochemical staining showed significantly lower expression of MAFbx, MuRF-1, UCP2, and UCP3 in the CO_2_-treated group compared with the control group, consistent with the quantitative real-time PCR results (Figs 5 and 6). MAFbx expression (± standard deviation) in the control group was 0.95±0.04, and that in the CO_2_-treated group was 0.17±0.04. MuRF-1 expression in the control group was 0.98±0.02, and that in the CO_2_-treated group was 0.26±0.07. UCP2 expression in the control group was 0.93±0.04 and that in the CO_2_-treated group was 0.20±0.11. UCP3 expression in the control group was 0.91±0.07, and that in the CO_2_-treated group was 0.16±0.06.

### 5. Cross‐sectional area of muscle

**Fig 7:**
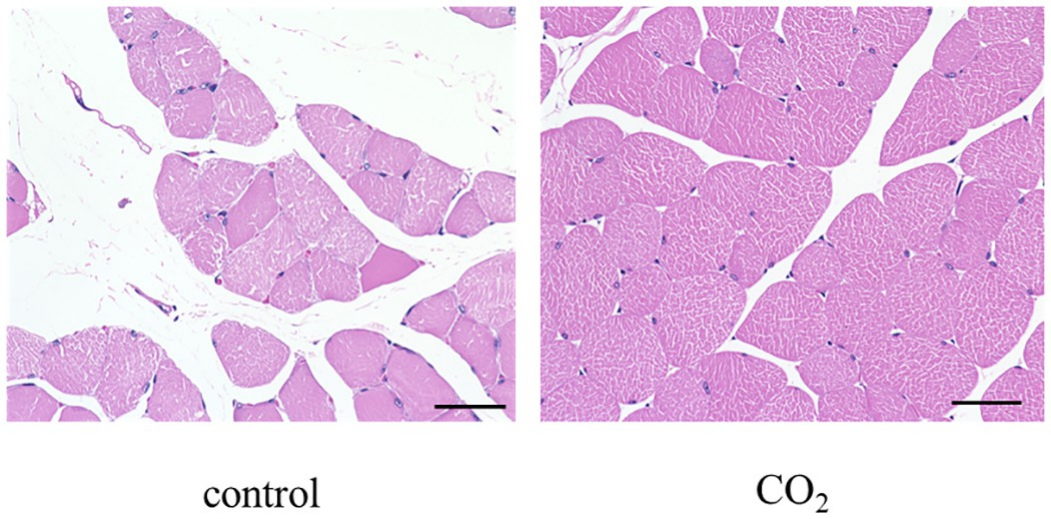
H&E staining of quadriceps muscle in the control group and CO_2_-treated group. The representive images are shown at ×400 magnification. Scale bar = 50μm.

**Fig 8:**
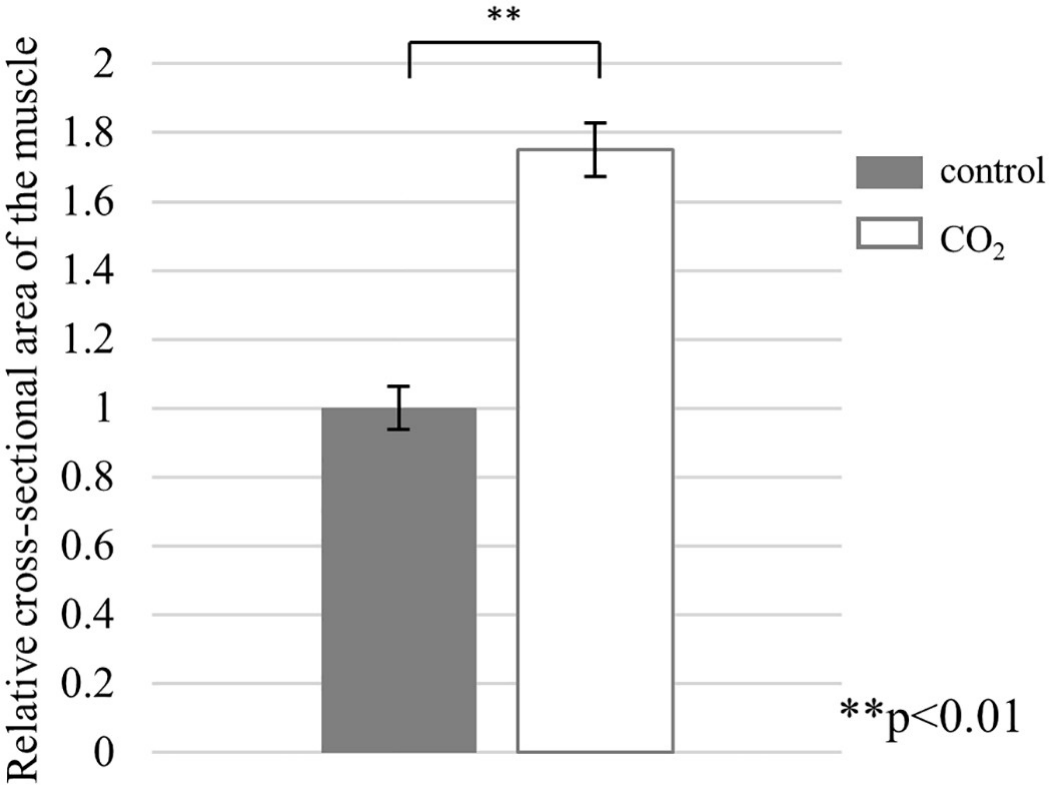
The relative cross-sectional area of quadriceps muscle in the control group (n = 7) and CO_2_-treated group (n = 7) (**P<0.01). The relative cross‐sectional area of the muscle (mean ± standard deviation) of the control group was 1.00 ± 0.17, and that of CO_2_-treated group was 1.75 ± 0.21.

Skeletal muscle atrophy caused a decrease in the myofiber area of the muscle tissue. At the end of the experiment, the relative cross‐sectional area of the muscle (mean ± standard deviation) of the control group was 1.00 ± 0.17, and that of the CO_2_-treated group was 1.75 ± 0.21. Furthermore, the relative cross‐sectional area of the muscle of the CO_2_-treated group was significantly larger than that of the control group (Figs 7 and 8).

## Discussion

Cachexia is a term that has been used for a long time to describe a state of wasting due to poor nutrition [1, 2]. Fearon et al. proposed a cancer-specific definition of cachexia in 2011 [1]. In this definition, cancer cachexia is loss of skeletal muscle mass through a progressively catabolic state.

Previous studies have attempted approaches including nutritional therapy, exercise therapy, and pharmacotherapy. However, these have not yet been established as definitive treatments for cancer, including head and neck cancers [4, 29–31]. Grande AJ et al. examined exercise therapy in patients with lean body masses in a study of cancer cachexia in adults, similar to our study. However, they concluded that the results were highly unclear. Because many cancer patients have impaired cardiac function or advanced anemia, these limitations could make it difficult for them to undertake appropriate exercise [30]. Furthermore, cancer cachexia is associated with increased side effects of chemotherapy and radiotherapy and increased complications associated with surgery, as well as a worse prognosis, including in head and neck cancer [3, 32].

CO_2_ therapy has been indicated for various diseases such as heart disease and skin conditions. The therapeutic effects of CO_2_ are mediated by an increase in blood flow and microcirculation, nitric oxide-dependent neocapillary formation, and increased partial pressure of oxygen in the local tissue, known as the Bohr effect. We have developed a new transcutaneous CO_2_ application system using 100% CO_2_ gas and a transdermal CO_2_ absorption-enhancing hydrogel [16]. This treatment has been used previously in several animal models, and it can increase local blood flow, prevent muscle atrophy and contraction, and recover muscle damage [18–23]. Oe et al. demonstrated similar changes to those that occur within muscles when aerobic exercise is performed [23]. And we have shown that the transcutaneous application of CO_2_ to the human neck and lower extremities can improve blood flow [17]. However, the effect of transcutaneous application of CO_2_ on cancer-related skeletal muscle atrophy is still unknown.

Skeletal muscle mass is maintained by a balance between the processes of protein synthesis and proteolysis. In cancer cachexia, this balance shifts to a catabolic state, leading to skeletal muscle atrophy [6]. It is known that many intracellular signals are activated by inflammatory mediators (such as cytokines) and tumor-derived factors (such as proteolysis-inducing factors). These factors activate forkhead family transcription factors, which enable transcription of genes encoding ubiquitin ligases [5]. The ubiquitin–proteasome pathway is known to be the primary proteolytic pathway in cancer cachexia and is induced by the E3 ubiquitin ligases [8, 9]. MAFbx and MuRF-1 are known E3 ubiquitin ligases specific to skeletal muscle and are often used as indicators of the muscle atrophy [6–11]. Proteasomes recognize and degrade ubiquitinated proteins. The expression of MAFbx and MuRF-1 are also known to be upregulated in skeletal muscle, as shown in various animal carcinoma models [8–11].

UCPs are identified as carriers of proton ions between the mitochondrial inner membrane and the mitochondrial matrix. When UCPs are increased, ATP synthesis is inhibited and is dissipated as heat. It is known that UCP2 is expressed in all tissues, including skeletal muscle, and UCP3 expression is present in skeletal and cardiac muscle [12]. In the skeletal muscle of a cancer-bearing animal model, it has been suggested that a decrease in mitochondrial ATP synthesis might occur due to the upregulation of UCPs [15]. In fact, Busquets et al. and Constantinou et al. demonstrated that a cancer-bearing rat model exhibited significantly more pronounced skeletal muscle atrophy compared with the control group rats, and the expression of UCP2 and UCP3 in skeletal muscles was significantly upregulated [13, 14]. Based on the above, in cancer cachexia, the activation of the ubiquitin–proteasome pathway and UCPs results in muscle atrophy. Additionally, the upregulation of UCPs could potentially lead to the downregulation of mitochondrial ATP synthesis.

In this study, we applied the transcutaneous application of CO_2_ to cancer-related skeletal muscle atrophy in vivo. We hypothesized that if the skeletal muscle atrophy improves, these four factors would be downregulated in skeletal muscle in the CO_2_-treated group. Skeletal muscle mass significantly differed between the control and CO_2_-treated group in the measurement of fat-free body mass by bioimpedance spectroscopy. Skeletal muscle atrophy was significantly suppressed in the cross‐sectional area analysis of muscle in the CO_2_-treated group. The mRNA expression of *MAFbx*, *MuRF-1*, *UCP2*, and *UCP3* was also predominantly downregulated in the CO_2_-treated group. Additionally, the expression of these four factors in the CO_2_-treated group was lower compared with that in control group by IHC staining. This indicated that the transcutaneous application of CO_2_ to skeletal muscle suppresses muscle atrophy through the downregulation of the ubiquitin–proteasome pathway and UCPs. This result is consistent with previous reports [7–11]. Furthermore, it was demonstrated that inhibiting the ubiquitin–proteasome pathway through the transcutaneous application of CO_2_ can suppress catabolism within the muscle tissue. It was also demonstrated that UCP2 and UCP3 expression can be decreased in skeletal muscle atrophy, suggesting a increase in ATP synthesis within the mitochondria, consistent with previous reports [13, 15]. Therefore, this mechanism may be considered to bring about an improvement in muscle atrophy through the enhancement of the expression of the four factors and the activation of the mitochondria pathway induced by CO_2_. However, t it has been reported that the expression of UCP3 was significantly increased, but UCP2 expression was not significantly increased in the skeletal muscle of cancer-bearing mice [14, 15, 33]. This result differs from those in our study. Furthermore, this study has some limitations. First, it is difficult to conduct experiments under pair-feeding conditions at our institute. Even if we could conduct experiments under pair-feeding conditions and maintain the same conditions such as food consumption, liquid consumption, and activity, the effects of cancer cachexia might vary widely among individuals. Second, we demonstrated that transcutaneous application of CO_2_ to skeletal muscle increased the number of mitochondria [18], but it is unclear whether this increase leads to elevated ATP synthesis. However, Vohwinkel et al. and Ceco et al. demonstrated that the potential biological effects of CO_2_ might also be related to cancer cachexia [34, 35], but in this study, the relationship was unclear. Third, we should focus on myogenesis-related factors as well as muscle atrophy factors in the future.

## Conclusions

In conclusion, we suggested that transcutaneous CO_2_ application to skeletal muscle suppresses the skeletal muscle atrophy of oral squamous cell cancer-bearing mice. These effects are thought to be due to inhibition of the ubiquitin–proteasome pathway in skeletal muscle, and the promotion of ATP synthesis might be related also. Due to its affordability and non-invasive nature, the CO_2_ treatment is easily applicable to humans. Therefore, it could be a preferred treatment option for patients with cancer cachexia. Additional research should be conducted to validate our findings.

## Supporting information

S1 Data (XLSX)
